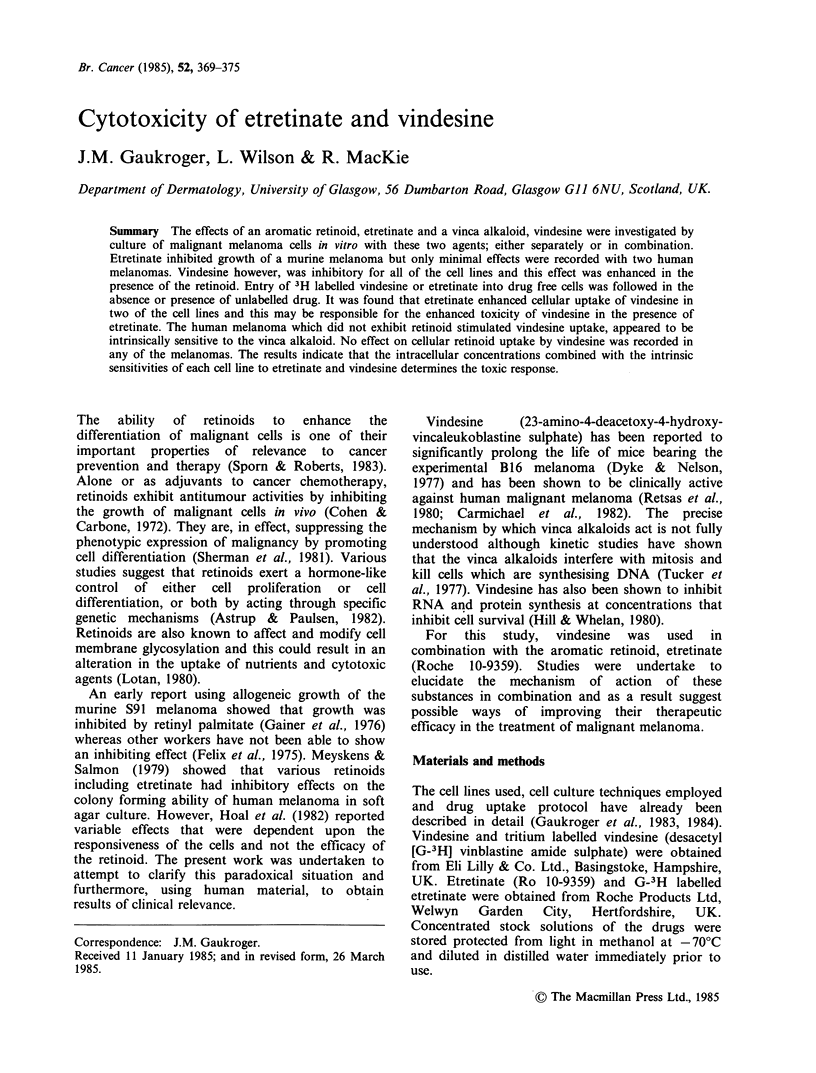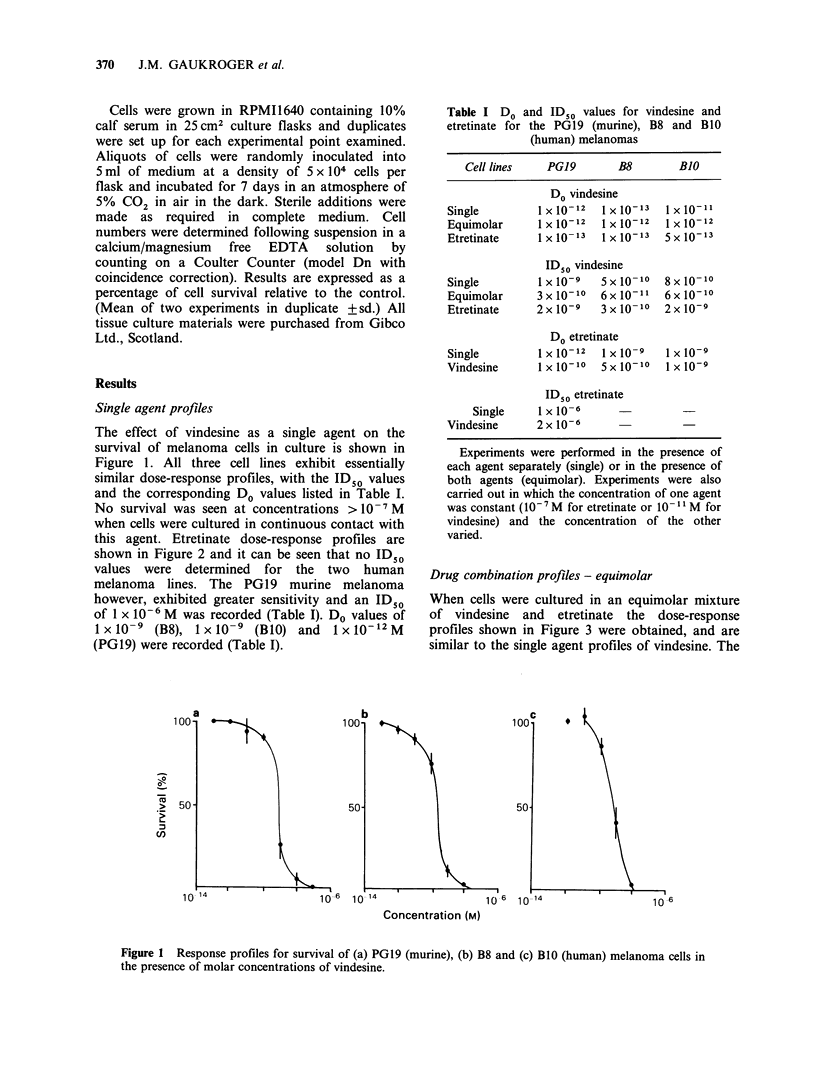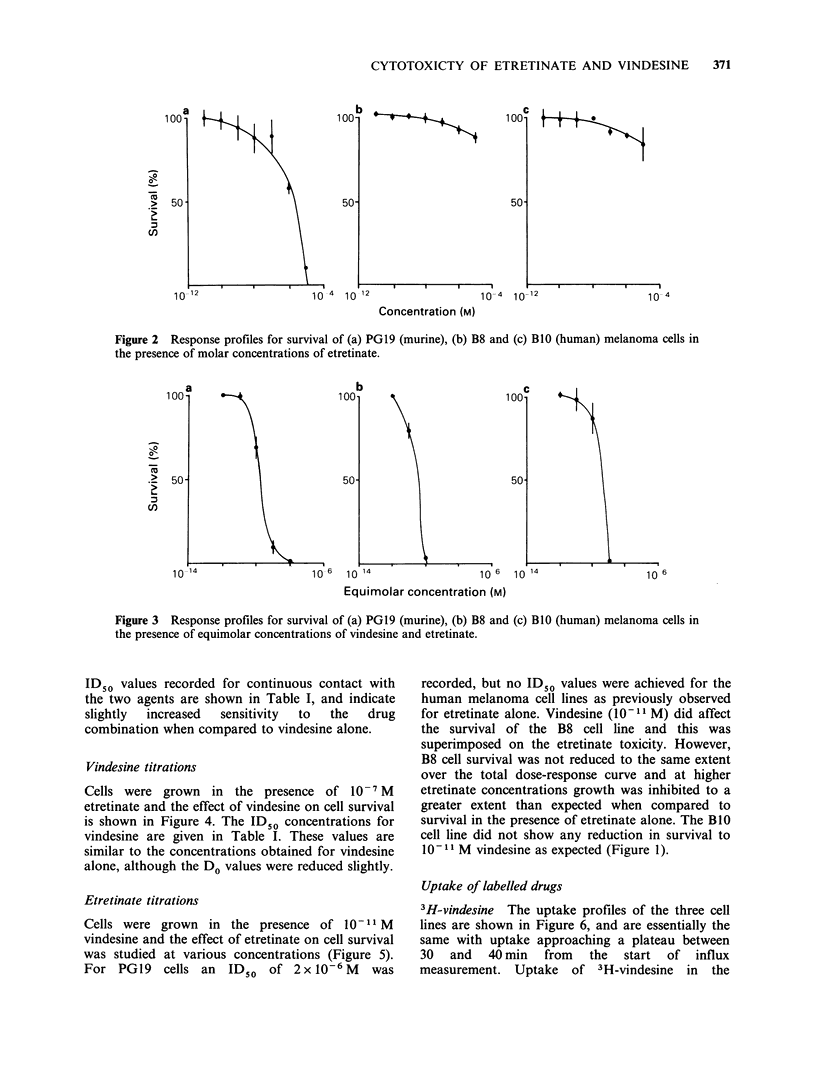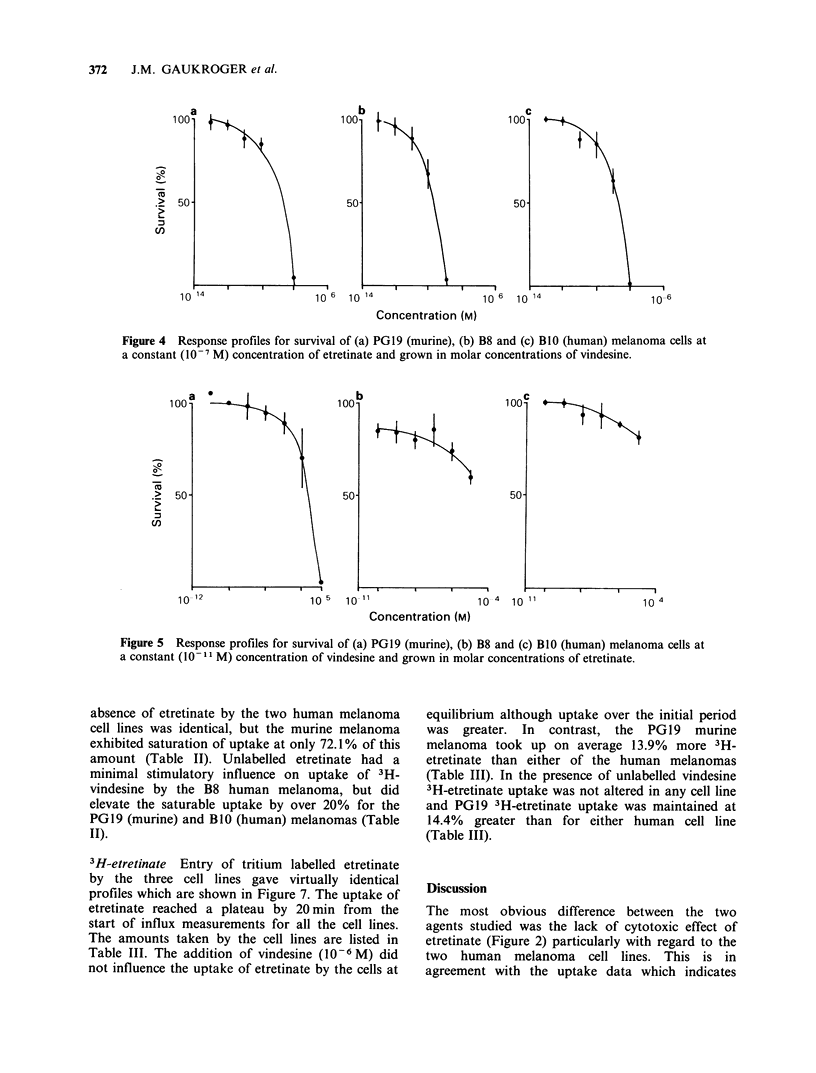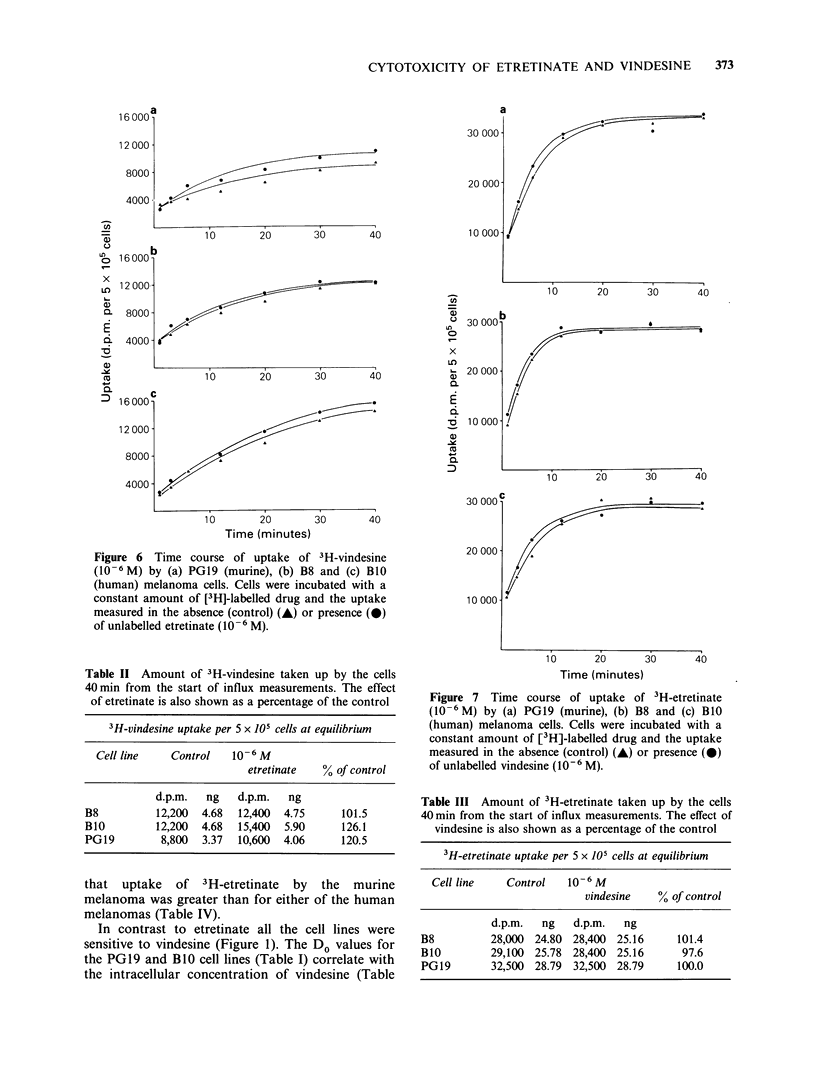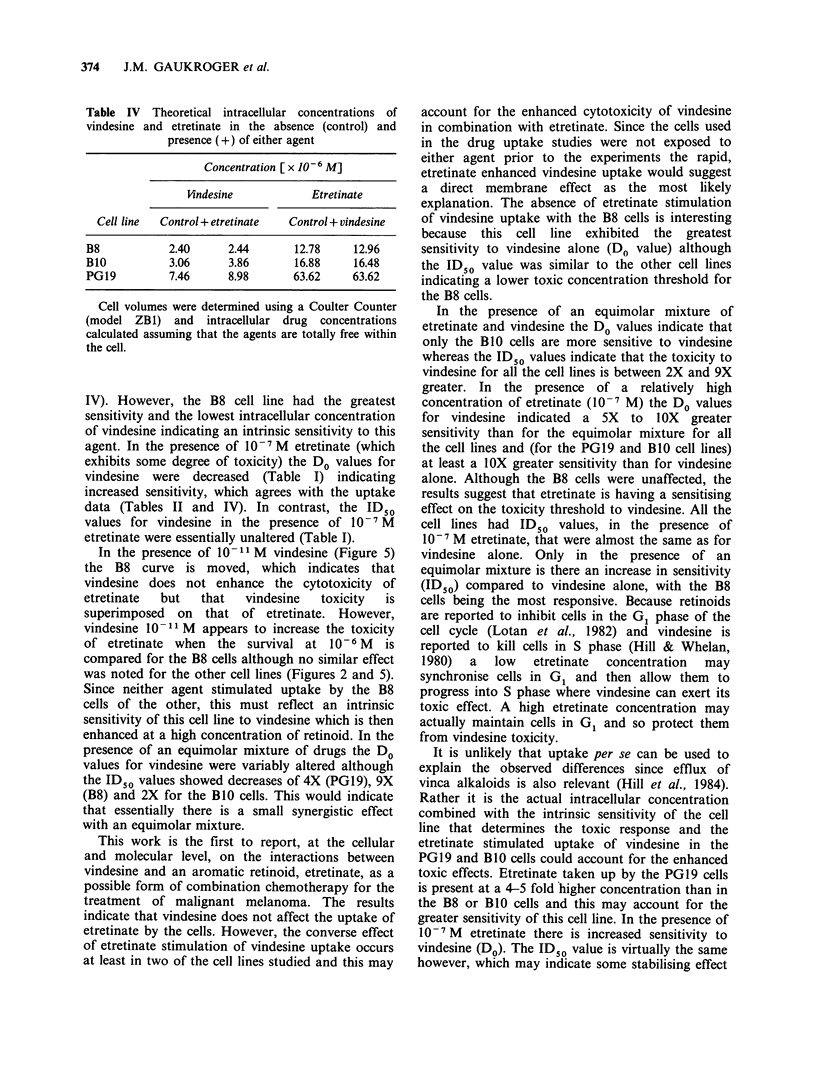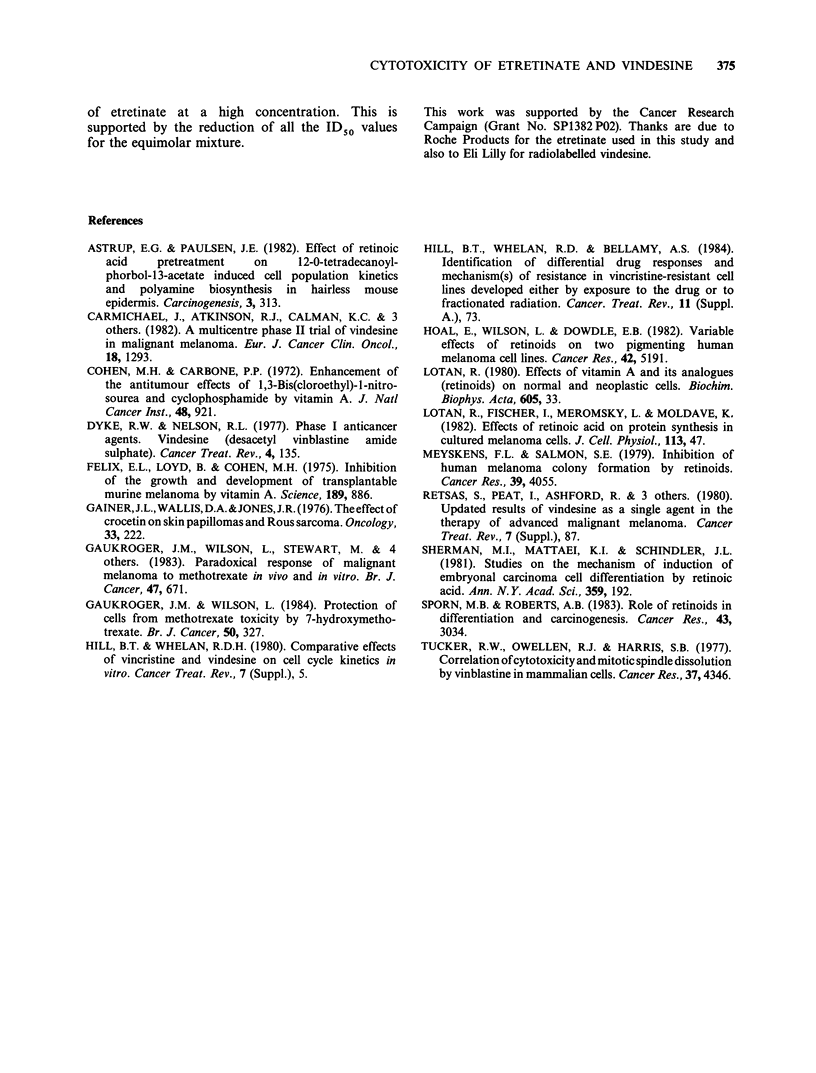# Cytotoxicity of etretinate and vindesine.

**DOI:** 10.1038/bjc.1985.203

**Published:** 1985-09

**Authors:** J. M. Gaukroger, L. Wilson, R. MacKie

## Abstract

The effects of an aromatic retinoid, etretinate and a vinca alkaloid, vindesine were investigated by culture of malignant melanoma cells in vitro with these two agents; either separately or in combination. Etretinate inhibited growth of a murine melanoma but only minimal effects were recorded with two human melanomas. Vindesine however, was inhibitory for all of the cell lines and this effect was enhanced in the presence of the retinoid. Entry of 3H labelled vindesine or etretinate into drug free cells was followed in the absence or presence of unlabelled drug. It was found that etretinate enhanced cellular uptake of vindesine in two of the cell lines and this may be responsible for the enhanced toxicity of vindesine in the presence of etretinate. The human melanoma which did not exhibit retinoid stimulated vindesine uptake, appeared to be intrinsically sensitive to the vinca alkaloid. No effect on cellular retinoid uptake by vindesine was recorded in any of the melanomas. The results indicate that the intracellular concentrations combined with the intrinsic sensitivities of each cell line to etretinate and vindesine determines the toxic response.


Br. Cancer (1985), 52, 369–375

### Cytotoxicity of etretinate and vindesine

J.M. Gaukroger, L. Wilson & R. MacKie

*Department of Dermatology, University of Glasgow, 56 Dumbarton Road, Glasgow G11 6NU, Scotland, UK.*

**Summary** The effects of an aromatic retinoid, etretinate and a vinca alkaloid, vindesine were investigated by culture of malignant melanoma cells *in vitro* with these two agents; either separately or in combination. Etretinate inhibited growth of a murine melanoma but only minimal effects were recorded with two human melanomas. Vindesine however, was inhibitory for all of the cell lines and this effect was enhanced in the presence of the retinoid. Entry of $^{3}H$ labelled vindesine or etretinate into drug free cells was followed in the absence or presence of unlabelled drug. It was found that etretinate enhanced cellular uptake of vindesine in two of the cell lines and this may be responsible for the enhanced toxicity of vindesine in the presence of etretinate. The human melanoma which did not exhibit retinoid stimulated vindesine uptake, appeared to be intrinsically sensitive to the vinca alkaloid. No effect on cellular retinoid uptake by vindesine was recorded in any of the melanomas. The results indicate that the intracellular concentrations combined with the intrinsic sensitivities of each cell line to etretinate and vindesine determines the toxic response.

The ability of retinoids to enhance the differentiation of malignant cells is one of their important properties of relevance to cancer prevention and therapy (Sporn & Roberts, 1983). Alone or as adjuvants to cancer chemotherapy, retinoids exhibit antitumour activities by inhibiting the growth of malignant cells *in vivo* (Cohen & Carbone, 1972). They are, in effect, suppressing the phenotypic expression of malignancy by promoting cell differentiation (Sherman *et al.*, 1981). Various studies suggest that retinoids exert a hormone-like control of either cell proliferation or cell differentiation, or both by acting through specific genetic mechanisms (Astrup & Paulsen, 1982). Retinoids are also known to affect and modify cell membrane glycosylation and this could result in an alteration in the uptake of nutrients and cytotoxic agents (Lotan, 1980).

An early report using allogeneic growth of the murine S91 melanoma showed that growth was inhibited by retinyl palmitate (Gainer *et al.*, 1976) whereas other workers have not been able to show an inhibiting effect (Felix *et al.*, 1975). Meyskens & Salmon (1979) showed that various retinoids including etretinate had inhibitory effects on the colony forming ability of human melanoma in soft agar culture. However, Hoal *et al.* (1982) reported variable effects that were dependent upon the responsiveness of the cells and not the efficacy of the retinoid. The present work was undertaken to attempt to clarify this paradoxical situation and furthermore, using human material, to obtain results of clinical relevance.

Correspondence: J.M. Gaukroger.
Received 11 January 1985; and in revised form, 26 March 1985.

Vindesine (23-amino-4-deacetoxy-4-hydroxy-vincaleukoblastine sulphate) has been reported to significantly prolong the life of mice bearing the experimental B16 melanoma (Dyke & Nelson, 1977) and has been shown to be clinically active against human malignant melanoma (Retsas *et al.*, 1980; Carmichael *et al.*, 1982). The precise mechanism by which vinca alkaloids act is not fully understood although kinetic studies have shown that the vinca alkaloids interfere with mitosis and kill cells which are synthesising DNA (Tucker *et al.*, 1977). Vindesine has also been shown to inhibit RNA and protein synthesis at concentrations that inhibit cell survival (Hill & Whelan, 1980).

For this study, vindesine was used in combination with the aromatic retinoid, etretinate (Roche 10-9359). Studies were undertake to elucidate the mechanism of action of these substances in combination and as a result suggest possible ways of improving their therapeutic efficacy in the treatment of malignant melanoma.

### Materials and methods

The cell lines used, cell culture techniques employed and drug uptake protocol have already been described in detail (Gaukroger *et al.*, 1983, 1984). Vindesine and tritium labelled vindesine (desacetyl [G-$^{3}H$] vinblastine amide sulphate) were obtained from Eli Lilly & Co. Ltd., Basingstoke, Hampshire, UK. Etretinate (Ro 10-9359) and G-$^{3}H$ labelled etretinate were obtained from Roche Products Ltd, Welwyn Garden City, Hertfordshire, UK. Concentrated stock solutions of the drugs were stored protected from light in methanol at −70°C and diluted in distilled water immediately prior to use.

© The Macmillan Press Ltd., 1985

Cells were grown in RPMI1640 containing 10% calf serum in 25 $cm^2$ culture flasks and duplicates were set up for each experimental point examined. Aliquots of cells were randomly inoculated into 5 ml of medium at a density of $5 \times 10^4$ cells per flask and incubated for 7 days in an atmosphere of 5% $CO_2$ in air in the dark. Sterile additions were made as required in complete medium. Cell numbers were determined following suspension in a calcium/magnesium free EDTA solution by counting on a Coulter Counter (model Dn with coincidence correction). Results are expressed as a percentage of cell survival relative to the control. (Mean of two experiments in duplicate $\pm$sd.) All tissue culture materials were purchased from Gibco Ltd., Scotland.

### Results

### *Single agent profiles*

The effect of vindesine as a single agent on the survival of melanoma cells in culture is shown in Figure 1. All three cell lines exhibit essentially similar dose-response profiles, with the $ID_{50}$ values and the corresponding $D_0$ values listed in Table I. No survival was seen at concentrations $>10^{-7}$ M when cells were cultured in continuous contact with this agent. Etretinate dose-response profiles are shown in Figure 2 and it can be seen that no $ID_{50}$ values were determined for the two human melanoma lines. The PG19 murine melanoma however, exhibited greater sensitivity and an $ID_{50}$ of $1 \times 10^{-6}$ M was recorded (Table I). $D_0$ values of $1 \times 10^{-9}$ (B8), $1 \times 10^{-9}$ (B10) and $1 \times 10^{-12}$ M (PG19) were recorded (Table I).

**Table I** $D_0$ and $ID_{50}$ values for vindesine and etretinate for the PG19 (murine), B8 and B10 (human) melanomas

| *Cell lines* | *PG19* | *B8* | *B10* |
|---|---|---|---|
| | $D_0$ vindesine | | |
| Single | $1 \times 10^{-12}$ | $1 \times 10^{-13}$ | $1 \times 10^{-11}$ |
| Equimolar | $1 \times 10^{-12}$ | $1 \times 10^{-12}$ | $1 \times 10^{-12}$ |
| Etretinate | $1 \times 10^{-13}$ | $1 \times 10^{-13}$ | $5 \times 10^{-13}$ |
| | $ID_{50}$ vindesine | | |
| Single | $1 \times 10^{-9}$ | $5 \times 10^{-10}$ | $8 \times 10^{-10}$ |
| Equimolar | $3 \times 10^{-10}$ | $6 \times 10^{-11}$ | $6 \times 10^{-10}$ |
| Etretinate | $2 \times 10^{-9}$ | $3 \times 10^{-10}$ | $2 \times 10^{-9}$ |
| | $D_0$ etretinate | | |
| Single | $1 \times 10^{-12}$ | $1 \times 10^{-9}$ | $1 \times 10^{-9}$ |
| Vindesine | $1 \times 10^{-10}$ | $5 \times 10^{-10}$ | $1 \times 10^{-9}$ |
| | $ID_{50}$ etretinate | | |
| Single | $1 \times 10^{-6}$ | — | — |
| Vindesine | $2 \times 10^{-6}$ | — | — |

Experiments were performed in the presence of each agent separately (single) or in the presence of both agents (equimolar). Experiments were also carried out in which the concentration of one agent was constant ($10^{-7}$ M for etretinate or $10^{-11}$ M for vindesine) and the concentration of the other varied.

### *Drug combination profiles – equimolar*

When cells were cultured in an equimolar mixture of vindesine and etretinate the dose-response profiles shown in Figure 3 were obtained, and are similar to the single agent profiles of vindesine. The

**Figure 1** Response profiles for survival of (a) PG19 (murine), (b) B8 and (c) B10 (human) melanoma cells in the presence of molar concentrations of vindesine.

**Figure 2** Response profiles for survival of (a) PG19 (murine), (b) B8 and (c) B10 (human) melanoma cells in the presence of molar concentrations of etretinate.

**Figure 3** Response profiles for survival of (a) PG19 (murine), (b) B8 and (c) B10 (human) melanoma cells in the presence of equimolar concentrations of vindesine and etretinate.

$ID_{50}$ values recorded for continuous contact with the two agents are shown in Table I, and indicate slightly increased sensitivity to the drug combination when compared to vindesine alone.

*Vindesine titrations*

Cells were grown in the presence of $10^{-7}$ M etretinate and the effect of vindesine on cell survival is shown in Figure 4. The $ID_{50}$ concentrations for vindesine are given in Table I. These values are similar to the concentrations obtained for vindesine alone, although the $D_0$ values were reduced slightly.

*Etretinate titrations*

Cells were grown in the presence of $10^{-11}$ M vindesine and the effect of etretinate on cell survival was studied at various concentrations (Figure 5). For PG19 cells an $ID_{50}$ of $2 \times 10^{-6}$ M was recorded, but no $ID_{50}$ values were achieved for the human melanoma cell lines as previously observed for etretinate alone. Vindesine ($10^{-11}$ M) did affect the survival of the B8 cell line and this was superimposed on the etretinate toxicity. However, B8 cell survival was not reduced to the same extent over the total dose-response curve and at higher etretinate concentrations growth was inhibited to a greater extent than expected when compared to survival in the presence of etretinate alone. The B10 cell line did not show any reduction in survival to $10^{-11}$ M vindesine as expected (Figure 1).

*Uptake of labelled drugs*

*$^3$H-vindesine* The uptake profiles of the three cell lines are shown in Figure 6, and are essentially the same with uptake approaching a plateau between 30 and 40 min from the start of influx measurement. Uptake of $^3$H-vindesine in the

**Figure 4** Response profiles for survival of (a) PG19 (murine), (b) B8 and (c) B10 (human) melanoma cells at a constant ($10^{-7}$ M) concentration of etretinate and grown in molar concentrations of vindesine.

**Figure 5** Response profiles for survival of (a) PG19 (murine), (b) B8 and (c) B10 (human) melanoma cells at a constant ($10^{-11}$ M) concentration of vindesine and grown in molar concentrations of etretinate.

absence of etretinate by the two human melanoma cell lines was identical, but the murine melanoma exhibited saturation of uptake at only 72.1% of this amount (Table II). Unlabelled etretinate had a minimal stimulatory influence on uptake of $^{3}$H-vindesine by the B8 human melanoma, but did elevate the saturable uptake by over 20% for the PG19 (murine) and B10 (human) melanomas (Table II).

*$^{3}$H-etretinate* Entry of tritium labelled etretinate by the three cell lines gave virtually identical profiles which are shown in Figure 7. The uptake of etretinate reached a plateau by 20 min from the start of influx measurements for all the cell lines. The amounts taken by the cell lines are listed in Table III. The addition of vindesine ($10^{-6}$ M) did not influence the uptake of etretinate by the cells at equilibrium although uptake over the initial period was greater. In contrast, the PG19 murine melanoma took up on average 13.9% more $^{3}$H-etretinate than either of the human melanomas (Table III). In the presence of unlabelled vindesine $^{3}$H-etretinate uptake was not altered in any cell line and PG19 $^{3}$H-etretinate uptake was maintained at 14.4% greater than for either human cell line (Table III).

### Discussion

The most obvious difference between the two agents studied was the lack of cytotoxic effect of etretinate (Figure 2) particularly with regard to the two human melanoma cell lines. This is in agreement with the uptake data which indicates

**Figure 6** Time course of uptake of $^3$H-vindesine ($10^{-6}$ M) by (a) PG19 (murine), (b) B8 and (c) B10 (human) melanoma cells. Cells were incubated with a constant amount of [$^3$H]-labelled drug and the uptake measured in the absence (control) (▲) or presence (●) of unlabelled etretinate ($10^{-6}$ M).

**Table II** Amount of $^3$H-vindesine taken up by the cells 40 min from the start of influx measurements. The effect of etretinate is also shown as a percentage of the control

| *$^3$H-vindesine uptake per 5 × $10^5$ cells at equilibrium* | | | | | |
|---|---|---|---|---|---|
| *Cell line* | *Control* | | *$10^{-6}$ M etretinate* | | *% of control* |
| | d.p.m. | ng | d.p.m. | ng | |
| B8 | 12,200 | 4.68 | 12,400 | 4.75 | 101.5 |
| B10 | 12,200 | 4.68 | 15,400 | 5.90 | 126.1 |
| PG19 | 8,800 | 3.37 | 10,600 | 4.06 | 120.5 |

that uptake of $^3$H-etretinate by the murine melanoma was greater than for either of the human melanomas (Table IV).

In contrast to etretinate all the cell lines were sensitive to vindesine (Figure 1). The $D_0$ values for the PG19 and B10 cell lines (Table I) correlate with the intracellular concentration of vindesine (Table

**Figure 7** Time course of uptake of $^3$H-etretinate ($10^{-6}$ M) by (a) PG19 (murine), (b) B8 and (c) B10 (human) melanoma cells. Cells were incubated with a constant amount of [$^3$H]-labelled drug and the uptake measured in the absence (control) (▲) or presence (●) of unlabelled vindesine ($10^{-6}$ M).

**Table III** Amount of $^3$H-etretinate taken up by the cells 40 min from the start of influx measurements. The effect of vindesine is also shown as a percentage of the control

| *$^3$H-etretinate uptake per 5 × $10^5$ cells at equilibrium* | | | | | |
|---|---|---|---|---|---|
| *Cell line* | *Control* | | *$10^{-6}$ M vindesine* | | *% of control* |
| | d.p.m. | ng | d.p.m. | ng | |
| B8 | 28,000 | 24.80 | 28,400 | 25.16 | 101.4 |
| B10 | 29,100 | 25.78 | 28,400 | 25.16 | 97.6 |
| PG19 | 32,500 | 28.79 | 32,500 | 28.79 | 100.0 |

**Table IV** Theoretical intracellular concentrations of vindesine and etretinate in the absence (control) and presence (+) of either agent

| | *Concentration* [ $\times 10^{-6}$ *M*] | | | |
|---|---|---|---|---|
| | *Vindesine* | | *Etretinate* | |
| *Cell line* | *Control* | *+ etretinate* | *Control* | *+ vindesine* |
| B8 | 2.40 | 2.44 | 12.78 | 12.96 |
| B10 | 3.06 | 3.86 | 16.88 | 16.48 |
| PG19 | 7.46 | 8.98 | 63.62 | 63.62 |

Cell volumes were determined using a Coulter Counter (model ZB1) and intracellular drug concentrations calculated assuming that the agents are totally free within the cell.

IV). However, the B8 cell line had the greatest sensitivity and the lowest intracellular concentration of vindesine indicating an intrinsic sensitivity to this agent. In the presence of $10^{-7}$ M etretinate (which exhibits some degree of toxicity) the $D_0$ values for vindesine were decreased (Table I) indicating increased sensitivity, which agrees with the uptake data (Tables II and IV). In contrast, the $ID_{50}$ values for vindesine in the presence of $10^{-7}$ M etretinate were essentially unaltered (Table I).

In the presence of $10^{-11}$ M vindesine (Figure 5) the B8 curve is moved, which indicates that vindesine does not enhance the cytotoxicity of etretinate but that vindesine toxicity is superimposed on that of etretinate. However, vindesine $10^{-11}$ M appears to increase the toxicity of etretinate when the survival at $10^{-6}$ M is compared for the B8 cells although no similar effect was noted for the other cell lines (Figures 2 and 5). Since neither agent stimulated uptake by the B8 cells of the other, this must reflect an intrinsic sensitivity of this cell line to vindesine which is then enhanced at a high concentration of retinoid. In the presence of an equimolar mixture of drugs the $D_0$ values for vindesine were variably altered although the $ID_{50}$ values showed decreases of 4X (PG19), 9X (B8) and 2X for the B10 cells. This would indicate that essentially there is a small synergistic effect with an equimolar mixture.

This work is the first to report, at the cellular and molecular level, on the interactions between vindesine and an aromatic retinoid, etretinate, as a possible form of combination chemotherapy for the treatment of malignant melanoma. The results indicate that vindesine does not affect the uptake of etretinate by the cells. However, the converse effect of etretinate stimulation of vindesine uptake occurs at least in two of the cell lines studied and this may account for the enhanced cytotoxicity of vindesine in combination with etretinate. Since the cells used in the drug uptake studies were not exposed to either agent prior to the experiments the rapid, etretinate enhanced vindesine uptake would suggest a direct membrane effect as the most likely explanation. The absence of etretinate stimulation of vindesine uptake with the B8 cells is interesting because this cell line exhibited the greatest sensitivity to vindesine alone ($D_0$ value) although the $ID_{50}$ value was similar to the other cell lines indicating a lower toxic concentration threshold for the B8 cells.

In the presence of an equimolar mixture of etretinate and vindesine the $D_0$ values indicate that only the B10 cells are more sensitive to vindesine whereas the $ID_{50}$ values indicate that the toxicity to vindesine for all the cell lines is between 2X and 9X greater. In the presence of a relatively high concentration of etretinate ($10^{-7}$ M) the $D_0$ values for vindesine indicated a 5X to 10X greater sensitivity than for the equimolar mixture for all the cell lines and (for the PG19 and B10 cell lines) at least a 10X greater sensitivity than for vindesine alone. Although the B8 cells were unaffected, the results suggest that etretinate is having a sensitising effect on the toxicity threshold to vindesine. All the cell lines had $ID_{50}$ values, in the presence of $10^{-7}$ M etretinate, that were almost the same as for vindesine alone. Only in the presence of an equimolar mixture is there an increase in sensitivity ($ID_{50}$) compared to vindesine alone, with the B8 cells being the most responsive. Because retinoids are reported to inhibit cells in the $G_1$ phase of the cell cycle (Lotan *et al.*, 1982) and vindesine is reported to kill cells in S phase (Hill & Whelan, 1980) a low etretinate concentration may synchronise cells in $G_1$ and then allow them to progress into S phase where vindesine can exert its toxic effect. A high etretinate concentration may actually maintain cells in $G_1$ and so protect them from vindesine toxicity.

It is unlikely that uptake *per se* can be used to explain the observed differences since efflux of vinca alkaloids is also relevant (Hill *et al.*, 1984). Rather it is the actual intracellular concentration combined with the intrinsic sensitivity of the cell line that determines the toxic response and the etretinate stimulated uptake of vindesine in the PG19 and B10 cells could account for the enhanced toxic effects. Etretinate taken up by the PG19 cells is present at a 4–5 fold higher concentration than in the B8 or B10 cells and this may account for the greater sensitivity of this cell line. In the presence of $10^{-7}$ M etretinate there is increased sensitivity to vindesine ($D_0$). The $ID_{50}$ value is virtually the same however, which may indicate some stabilising effect of etretinate at a high concentration. This is supported by the reduction of all the $ID_{50}$ values for the equimolar mixture.

This work was supported by the Cancer Research Campaign (Grant No. SP1382 P02). Thanks are due to Roche Products for the etretinate used in this study and also to Eli Lilly for radiolabelled vindesine.

### References

ASTRUP, E.G. & PAULSEN, J.E. (1982). Effect of retinoic acid pretreatment on 12-0-tetradecanoyl-phorbol-13-acetate induced cell population kinetics and polyamine biosynthesis in hairless mouse epidermis. *Carcinogenesis,* **3,** 313.

CARMICHAEL, J., ATKINSON, R.J., CALMAN, K.C. & 3 others. (1982). A multicentre phase II trial of vindesine in malignant melanoma. *Eur. J. Cancer Clin. Oncol.,* **18,** 1293.

COHEN, M.H. & CARBONE, P.P. (1972). Enhancement of the antitumour effects of 1,3-Bis(cloroethyl)-1-nitro-sourea and cyclophosphamide by vitamin A. *J. Natl Cancer Inst.,* **48,** 921.

DYKE, R.W. & NELSON, R.L. (1977). Phase I anticancer agents. Vindesine (desacetyl vinblastine amide sulphate). *Cancer Treat. Rev.,* **4,** 135.

FELIX, E.L., LOYD, B. & COHEN, M.H. (1975). Inhibition of the growth and development of transplantable murine melanoma by vitamin A. *Science,* **189,** 886.

GAINER, J.L., WALLIS, D.A. & JONES, J.R. (1976). The effect of crocetin on skin papillomas and Rous sarcoma. *Oncology,* **33,** 222.

GAUKROGER, J.M., WILSON, L., STEWART, M. & 4 others. (1983). Paradoxical response of malignant melanoma to methotrexate *in vivo* and *in vitro*. *Br. J. Cancer,* **47,** 671.

GAUKROGER, J.M. & WILSON, L. (1984). Protection of cells from methotrexate toxicity by 7-hydroxymetho-trexate. *Br. J. Cancer,* **50,** 327.

HILL, B.T. & WHELAN, R.D.H. (1980). Comparative effects of vincristine and vindesine on cell cycle kinetics *in vitro*. *Cancer Treat. Rev.,* **7** (Suppl.), 5.

HILL, B.T., WHELAN, R.D. & BELLAMY, A.S. (1984). Identification of differential drug responses and mechanism(s) of resistance in vincristine-resistant cell lines developed either by exposure to the drug or to fractionated radiation. *Cancer. Treat. Rev.,* **11** (Suppl. A.), 73.

HOAL, E., WILSON, L. & DOWDLE, E.B. (1982). Variable effects of retinoids on two pigmenting human melanoma cell lines. *Cancer Res.,* **42,** 5191.

LOTAN, R. (1980). Effects of vitamin A and its analogues (retinoids) on normal and neoplastic cells. *Biochim. Biophys. Acta,* **605,** 33.

LOTAN, R., FISCHER, I., MEROMSKY, L. & MOLDAVE, K. (1982). Effects of retinoic acid on protein synthesis in cultured melanoma cells. *J. Cell. Physiol.,* **113,** 47.

MEYSKENS, F.L. & SALMON, S.E. (1979). Inhibition of human melanoma colony formation by retinoids. *Cancer Res.,* **39,** 4055.

RETSAS, S., PEAT, I., ASHFORD, R. & 3 others. (1980). Updated results of vindesine as a single agent in the therapy of advanced malignant melanoma. *Cancer Treat. Rev.,* **7** (Suppl.), 87.

SHERMAN, M.I., MATTAEI, K.I. & SCHINDLER, J.L. (1981). Studies on the mechanism of induction of embryonal carcinoma cell differentiation by retinoic acid. *Ann. N.Y. Acad. Sci.,* **359,** 192.

SPORN, M.B. & ROBERTS, A.B. (1983). Role of retinoids in differentiation and carcinogenesis. *Cancer Res.,* **43,** 3034.

TUCKER, R.W., OWELLEN, R.J. & HARRIS, S.B. (1977). Correlation of cytotoxicity and mitotic spindle dissolution by vinblastine in mammalian cells. *Cancer Res.,* **37,** 4346.